# δ-ALA-D activity is a reliable marker for oxidative stress in bone marrow transplant patients

**DOI:** 10.1186/1471-2407-9-138

**Published:** 2009-05-08

**Authors:** Thissiane L Gonçalves, Dalila M Benvegnú, Gabriela Bonfanti, Andressa V Frediani, João Batista T Rocha

**Affiliations:** 1 Departamento de Química, CCNE, Universidade Federal de Santa Maria, Santa Maria, R.S., Brazil; 2 Departamento de Análises Clínicas e Toxicológicas, CCS, Universidade Federal de Santa Maria, Santa Maria, RS, Brazil

## Abstract

**Background:**

Bone marrow transplantation (BMT) is often used in the treatment of various diseases. Before BMT, patients are submitted to a conditioning regimen (CR), which consists of the administration of high doses of chemotherapy. The action of many cytostatic drugs involves the overproduction of reactive oxygen species, which together with inadequate antioxidant protection can lead to oxidative stress and this has been implicated in the etiology of various diseases. The objectives of this study were to look for evidence of oxidative stress and also to analyze δ-Aminolevulinato dehydratase (δ-ALA-D) activity as a possible marker of oxidative stress in autologous and allogeneic BMT patients.

**Methods:**

Lipid peroxidation, vitamin C and thiol group levels as well as catalase, superoxide dismutase and δ-ALA-D activity were determined in 37 healthy controls, 13 patients undergoing autologous peripheral blood stem cell transplantation and 24 patients undergoing allogeneic BMT.

**Results:**

We found that patients presented signs of oxidative stress before they were submitted to BMT, during CR and up to 20 days after BMT. There was a decrease in enzymatic and non enzymatic antioxidant defenses, in δ-ALA-D activity, and an increase in lipoperoxidation in the blood of both patient groups.

**Conclusion:**

This study has indicated that autologous and allogeneic BMT are associated with oxidative stress. Moreover, blood δ-ALA-D activity seems to be an additional biomarker of oxidative stress in BMT patients.

## Background

Bone marrow transplantation (BMT) is a therapeutic method used in various malignant, hematologic, immunologic, and genetic diseases [1]. Allogeneic transplantation involves the transfer of marrow from a donor to another person, and one of the main problems in this procedure is the graft-versus-host disease [2]. Allogeneic transplantations are preferred for patients with leukemias or myeloproliferative diseases [3] whereas autologous transplantations are generally performed in patients with multiple myeloma (MM) or lymphoma [4,5]. Autologous BMT involves the use of the patient's own marrow to reestablish hematopoietic cell function after the administration of high-dose chemotherapy. In this case the stem cells can be taken from the patient's bone marrow or peripheral blood [2]. Peripheral blood stem cell transplantation (PBSCT) with collection by apheresis has replaced bone marrow as a source of hematopoietic stem cells for autologous transplantation, because this type of collection results in a more rapid hematopoietic recovery [5,6]. In contrast, in allogeneic transplantation, bone marrow continues to be the principal source of cells [7].

Unlike allogeneic transplantation, autologous transplantation is a more simple procedure that can be performed safely in older patients, because there is no risk of graft-versus-host disease as a complication [2]. However, the high incidence of relapse as a consequence of reinfusion of malignant stem cells is an important problem in autologous BMT [5].

BMT is always associated with intense and variable organic toxicity and with severe and prolonged myelosuppression, which give rise to a period of high vulnerability in which patients may develop complications. Administration of high doses of chemotherapy with or without total body irradiation (TBI) is a feature of BMT protocols known as the CR. These regimens are designed to abate the underlying malignant cells in the autologous BMT, to cause immunosuppression in order to avoid destruction of the allograft, and to destroy any residual cancer cells in allogeneic BMT [2].

The production of free radicals and the deficiency of antioxidants can to lead to a condition known as oxidative stress, which can be associated with serious complications in different types of transplantation [8]. Chemotherapy and radiotherapy are associated with the generation of large amounts of reactive oxygen/nitrogen species and depletion of antioxidants (vitamins A, C and E, reduced glutathione-GSH) and with an increase in plasma lipid hydroperoxides and TBARS [9-16]. Sulfhydryl groups (-SH) play important roles in a variety of cell activities and they can be easily modified by oxidants or alkylating agents [17]. In fact, -SH group content can be depleted after disproportional oxidation by free radicals or after formation of adducts with reactive chemicals [18].

δ-Aminolevulinate dehydratase (δ-ALA-D) is a sulfhydryl-containing enzyme [19,20] that catalyses the synthesis of tetrapyrrolic compounds such as billins and hemes. Consequently, δ-ALA-D is essential for aerobic organisms [21,22]. Recently, data from literature and from our laboratory have indicated that δ-ALA-D is sensitive to situations associated with oxidative stress [23-27]. Furthermore, enzyme inhibition can lead to accumulation of its substrate, 5-aminolevulinate (ALA), in the blood, which in turn can intensify oxidative stress by generating carbon-centered reactive species or by releasing iron from proteins such as ferritin [28].

The BMT procedure is toxic and excessive free radical production has been implicated in the action of many cytostatic drugs [29], but little is known about how cytostatic drugs affect the antioxidative system in human beings and only a few studies have been performed in the clinical setting [10]. In this context, the objective of this study was to look for further evidence of oxidative stress and also to analyze δ-ALA-D activity as a possible marker of oxidative stress in autologous and allogeneic BMT patients as well as to compare these two procedure in order to determine whether either of them could be less toxic to patients, when markers of oxidative stress are used as the end point of toxicity.

## Methods

### Subjects

Altogether, 37 patients: 13 undergoing autologous PBSCT, 03 female and 10 male, mean age 48.15 ± 12.67 years, 24 undergoing allogeneic BMT, 12 female and 12 male, mean age 34.13 ± 15.41 and 37 controls (matched by age and sex with the patients) were included in the investigation (samples were obtained from March 2007 to March 2008). The patients were under treatment in the 'Hospital Universitário' from the 'Universidade Federal de Santa Maria' (HUSM), RS, Brazil. They were treated with autologous PBSCT or allogeneic BMT which were preceded by CR (see table 1).

**Table 1:** Characteristics of patients

Number of patients (n = 37)	Autologous PBSCT group II (n = 13)	Allogeneic BMT group III (n = 24)
Mean age (years)	48.15 ± 12.67^b^	36.00 ± 16.08
Male	10	12
Female	03^b^	12
Diagnosis		
Multiple myeloma	03	01
Lymphoma Hodgkin	05	03
Lymphoma No Hodgkin	02	02
Leukemia		
Leukemia Acute myeloid	01	02
Leukemia Chronic myeloid	00	07
Leukemia Acute lymphoblastic	01	01
Leukemia Chronic lymphoid	00	01
Myelodysplasia	00	05
Aplastic anemia	00	02
Tumor Ewing	01	00
Conditioning regimen		
BuCy 120	01	13
BuCy 200	01	00
M-200	04	01
CBV	04	00
FluCy	00	05
BEAM	02	00
CyTBI	01	03
another	00	02

The CR patients were divided according to diseases. M-200: melphalan (200 mg/m^2^) given only 1 day (day minus 3) before the transplantation for multiple myeloma in autologous peripheral blood stem cell transplantation (PBSCT) or allogeneic BMT. CBV: cyclophosphamide (1500 mg/m^2^) given 4 consecutive days starting 6 days before the transplantation (days minus 6 to minus 3) + carmustine (BCNU – 450 mg/m^2^) given only 1 day before the transplantation (day minus 6) + etoposide (VP-16 – 250 mg/m^2^) given 3 consecutive days two times a day, starting 6 days before the transplantation (days minus 6 to minus 4) or BEAM: carmustine (BCNU – 300 mg/m^2^) given only 1 day before the transplantation (day minus 7) + etoposide (VP-16 – 200 mg/m^2^) given 4 consecutive days two times a day, starting 6 days before the transplantation (days minus 6 to minus 3) + cytarabine (100 mg/m^2^) given 4 consecutive days two times a day, starting 6 days before the transplantation (days minus 6 to minus 3) + melphalan (140 mg/m^2^) given only 1 day before the transplantation (day minus 6) for Hodgkin and non-Hodgkin lymphomas in autologous PBSCT. FluCy: fludarabine (30 mg/m^2^) + cyclophosphamide (300 mg/m^2^), both given 3 consecutive days, starting 4 days before the transplantation (days minus 4 to minus 2) for Hodgkin and non-Hodgkin lymphomas in allogeneic BMT. BuCy120: busulphan (1 mg/kg), given 4 consecutive days four times a day, starting 8 days before the transplantation (days minus 8 to minus 5) + cyclophosphamide (60 mg/kg), given 2 consecutive days, starting 4 days before the transplantation (days minus 4 to minus 3) for acute and chronic myeloid leukemia and syndrome mielodysplastic in autologous PBSCT or allogeneic BMT. BuCy200: busulphan (1 mg/kg), given 3 consecutive days four times a day, starting 9 days before the transplantation (days minus 9 to minus 7) + cyclophosphamide (50 mg/m^2^), given 4 consecutive days, starting 6 days before the transplantation (days minus 6 to minus 3) for aplastic anemia in allogeneic BMT. CyTBI: cyclophosphamide (60 mg/m^2^), given 2 consecutive days, starting 7 days before the transplantation (days minus 7 to minus 6) + total body irradiation, carried out on 4 consecutive days, starting 4 days before the transplantation (days minus 4 to minus 2, three times a day and day minus 1, two times a day) for acute and chronic lymphoid leukemia in autologous PBSCT or allogeneic BMT. All patients had 2 rest days (without chemotherapy) before the transplantation (days minus 2 and minus 1), except FluCy and CyTBI CR patients, that had only one rest day (day minus 1 and minus 5, respectively).

^b ^Significantly different between groups II (autol.) and III (allog.).

The present study was approved by the Human Ethical Committee of the Universidade Federal de Santa Maria, protocol number 0152.0243.000-06. All persons gave their informed consent prior to their inclusion in the study.

The subjects included in the study were classified in three groups: Group I – Control: healthy volunteers of HUSM Blood Bank served as control group and they were receiving no treatment for any diseases; Group II (autol.): autologous PBSCT patients; Group III (allog.): allogeneic BMT patients.

Table 1 shows the characteristics of the patients included in this study.

In our study, the patients were significantly older in group II (autol.) than in group III (allog.), p < 0.05. This is in agreement with the literature, where 40 to 55 years is considered the oldest age for patients undergoing allogeneic transplantation and autologous transplantation is reported to be safer in older patients, because there is no risk of graft-versus-host disease as a complication [2]. In our study there were also fewer females in group II (autol.) than in group III (alog.), perhaps because the autologous procedure is utilized for treatment of diseases that are predominant in males, such as lymphomas. Some studies have shown a male predominance in this disease [30-32] as well as in myeloma [33].

### Sample collection

Blood (4 mL) was collected during routine examinations by venous arm puncture in EDTA vaccutainer tubes in the Hematology and Oncology Laboratory. The blood used in this study was the leftover of this 4 mL sample. Four samples of each patient were taken, the first before CR, the second during CR (the last day the patient received chemotherapy), the third 10 days after BMT and the fourth 20 days after BMT. Control group samples were the leftover blood of HUSM Blood Bank donors, which was collected on the same day of the matched patient. Only one sample was used from these volunteers (before CR, CR, 10 and 20 days after BMT, consequently, control values for each time point are from different subjects). The plasma and cells were separated by centrifugation at 1000 rpm for 12 min. Then a portion of the collected plasma and erythrocytes were stored for analysis at -20°C for less than 3 weeks. The other analyses were performed on the same day.

### Biochemical estimations

All biochemical assays were made in duplicates or triplicates, depending on the availability of samples.

### Thiobarbituric acid-reactive substances

Thiobarbituric acid-reactive substances (TBARS) assay measures the peroxidative damage to lipids that occurs by excessive ROS generation. Lipid peroxidation was estimated in plasma according to the method of Lappena et al. (2001), using 1% phosphoric acid and 0.6% thiobarbituric acid (TBA). The pink chromogen produced by reaction of TBA with malondialdehyde (MDA), was measured spectrophotometrically at 532 nm. The results were expressed as nmol TBARS/mL plasma, using MDA as standard [34].

### Catalase enzyme activity

Catalase (CAT) enzyme activity was measured by the method of Aebi (1984). Packed erythrocytes were hemolyzed by adding 100 volumes of distilled water, then, 20 μL of this hemolyzed sample was added to a cuvette and the reaction was started by the addition of 100 μL of freshly prepared 300 mM H_2_O_2 _in phosphate buffer (50 mM, pH 7.0) to give a final volume of 1 mL. The rate of H_2_O_2 _decomposition was measured spectrophotometrically at 240 nm during 120 s. The catalase activity was expressed as μmol H_2_O_2_/mL erythrocytes/min [35].

### Superoxide dismutase enzyme activity

Superoxide dismutase (SOD) activity was performed according to the method of Misra and Fridovich (1972). Briefly, epinephrine rapidly auto oxidizes at pH 10.5 producing adrenochrome, a pink colored product that can be detected at 480 nm. The addition of samples containing SOD inhibits epinephrine auto-oxidation. The inhibition rate was monitored during 150 s at intervals of 10 s. The amount of enzyme required to produce 50% inhibition at 25°C was defined as one unit of enzyme activity. The SOD activity was expressed as U/mL erythrocytes [36].

### Vitamin C

Plasma vitamin C (VIT C) was estimated as described by Galley et al. (1996) with some modifications by Jacqes-Silva et al. (2001). Plasma was precipitated with 1 volume of a cold 5% trichloroacetic acid solution followed by centrifugation. An aliquot of 300 μL of the supernatants were mixed with 2,4-dinitrophenylhydrazine (4.5 mg/mL) and 13.3% trichloroacetic acid and incubated for 3 h at 37°C. Then, 1 mL 65% sulfuric acid was added to the medium and the orange red compound was measured at 520 nm. The content of ascorbic acid was calculated using a standard curve (1.5 – 4.5 μmol/L ascorbic acid freshly prepared in sulfuric acid) and expressed as μg vit C/mL plasma [37,38].

### Protein thiol groups

Protein thiol groups (P-SH) were assayed in plasma by the method of Boyne and Ellman (1972) modified by Jacques-Silva et al. (2001), which consisted of the reduction of 5.5'-dithio(bis-nithrobenzoic) acid (DTNB) in 0.3 M phosphate buffer, pH 7.0, measured at 412 nm. Quantification of total protein-SH groups may indicate thiol status and may also indicate the general state of thiol-containing proteins and, indirectly, the redox state of the blood cells. A standard curve using glutathione was constructed in order to calculate the protein thiol groups. The results were expressed as nmol P-SH/mL plasma [39,38].

### Non protein thiol groups

Erythrocyte non protein thiol groups (NP-SH) were determined as described by Boyne and Ellman (1972) modified by Jacques-Silva et al. (2001). Red blood cell pellets (300 μL) obtained after centrifugation of whole blood were hemolyzed with 10% triton solution (100 μL) for 10 min. Then, the protein fraction was precipitated with 200 μL of 20% trichloroacetic acid followed by centrifugation. Quantification of non-protein thiols (NP-SH) may indicate 90% of GSH content in the blood [40], which is one of the most important reducing agents in different mammal cells [16]. The colorimetric assay was carried out in 1 M phosphate buffer, pH 7.4. A standard curve using glutathione was constructed in order to calculate non protein thiol groups. The NP-SH level was measured at 412 nm and expressed as nmol NP-SH/mL erythrocytes. [39,38].

### δ-Aminolevulinate dehydratase (δ-ALA-D) activity

δ-Aminolevulinate dehydratase (δ-ALA-D) activity was assayed in whole blood by the method of Berlin and Schaller [41] by measuring the rate of porphobilinogen (PBG) formation in 1 h at 37°C. The enzyme reaction was initiated after 10 min of pre-incubation of blood with 1 mM ZnCl_2_. The reaction was started by adding δ-aminolevulinic acid (ALA) to a final concentration of 4 mM in a phosphate buffered solution, and incubation was carried out for 1 h at 37°C and the reaction product was measured at 555 nm and expressed as nmol PBG/mL blood/h.

### Statistical analysis

Biochemical assay results were expressed as median (lower/upper quartiles) and ages were expressed as mean ± SD.

Since data had no homogeneity of variance, statistical analysis was performed using Kruskal-Wallis ANOVA followed by Mann-Whitney U test to compare the difference among the groups, Friedman ANOVA followed by Wilcoxon test to analyze changes in blood indices over time and Spearman correlation to analyze correlations between biochemical estimations. Test T was used to compare the difference in the ages and sex among the groups. A value of p < 0.05 was considered statistically significant.

## Results

### Lipid peroxidation

Plasma TBARS levels were significantly higher in group II (autol.) than group I (control), before CR, during CR and on days 10 and 20 after BMT, (p < 0.05). TBARS levels were higher in group III (allog.) than in group I (control), during CR and on days 10 and 20 after BMT, (p < 0.0001). Groups II and III were not significantly different from each other in TBARS levels. TBARS levels increased from before CR and during CR to on days 10 and 20 after BMT in group III (p < 0.05), (table 2).

**Table 2:** Plasma lipid peroxidation (TBARS) levels over time

Groups	Before CR	CR	Day 10 after BMT	Day 20 after BMT
Group I (control)	15.01(12.76/19.25)	14.40(11.66/16.06)	14.52(11.56/15.30)	15.02(12.55/16.58)
Group II (autol.)	21.12(17.76/29.70)^a^	21.89(17.60/26.40)^a^	20.46(15.45/32.21)^a^	19.36(15.62/27.61)^a^
Group III (allog.)	18.26(15.40/22.00)	19.14(16.60/23.10)^a^	29.23(21.34/40.72)^acd^	27.50(18.92/38.50)^acd^

CR: conditioning regimen; BMT: bone marrow transplantation; Units – TBARS: nmol TBARS/mL plasma. Data are expressed as median (lower/upper quartile). Group I (n = 37); Group II (n = 13); Group III (n = 24).

^a ^Significantly different from group I (control).

^c ^Significantly different from before CR.

^d ^Significantly different from CR.

### Vitamin C

Plasma vitamin C (VIT C) concentrations were significantly decreased in groups II (autol.) and III (allog.), before CR (p < 0.05), during CR and on days 10 and 20 after BMT (p < 0.005), when compared with group I (control). Groups II and III were not significantly different from each other in VIT C levels. In group II, VIT C levels decreased significantly from before CR to during CR and in group III from before CR to during CR and on days 10 and 20 after BMT (p < 0.05), (table 3).

**Table 3:** Plasma vitamin C (VIT C) levels over time

Groups	Before CR	CR	Day 10 after BMT	Day 20 after BMT
Group I (control)	22.75(16.92/26.40)	17.80(12.90/21.84)	17.80(15.40/22.13)	19.20(16.38/24.26)
Group II (autol.)	12.70(8.80/19.80)^a^	9.90(8.02/11.76)^ac^	7.21(5.33/8.00)^a^	9.72(6.53/10.82)^a^
Group III (allog.)	13.08(9.96/16.66)^a^	8.09(4.63/10.50)^ac^	7.50(6.48/12.50)^ac^	9.10(4.37/12.40)^ac^

CR: conditioning regimen; BMT: bone marrow transplantation; Units – vitamin C: μg/mL plasma.

Data are expressed as median (lower/upper quartile). Group I (n = 37); Group II (n = 13); Group III (n = 24).

^a ^Significantly different from group I (control).

^c ^Significantly different from before CR.

### Catalase

**Figure 1:**
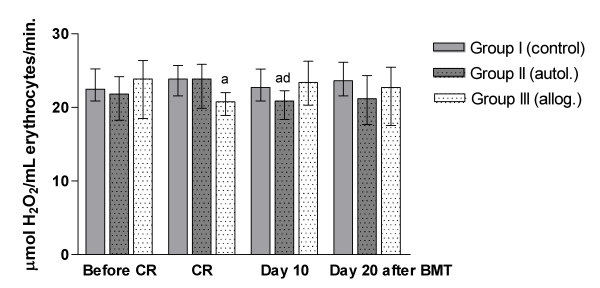
**Erythrocyte catalase (CAT) activity over time**. CR: conditioning regimen; BMT: bone marrow transplantation. Data are expressed as median (lower/upper quartile). Group I (n = 37); Group II (n = 13); Group III (n = 24).^a ^Significantly different from group I (control), ^d^significantly different from CR.

Erythrocyte catalase (CAT) activity was significantly lower in group II (autol.) than in group I (control) and on day 10 after BMT, (p < 0.05) and was also lower in group III (allog.) than in group I (control) during CR (p < 0.01). Groups II and III were not significantly different from each other in erythrocyte CAT activity. In group II, CAT activity decreased from during CR to day 10 after BMT (p < 0.05), (Fig. 1).

### Superoxide dismutase

**Figure 2:**
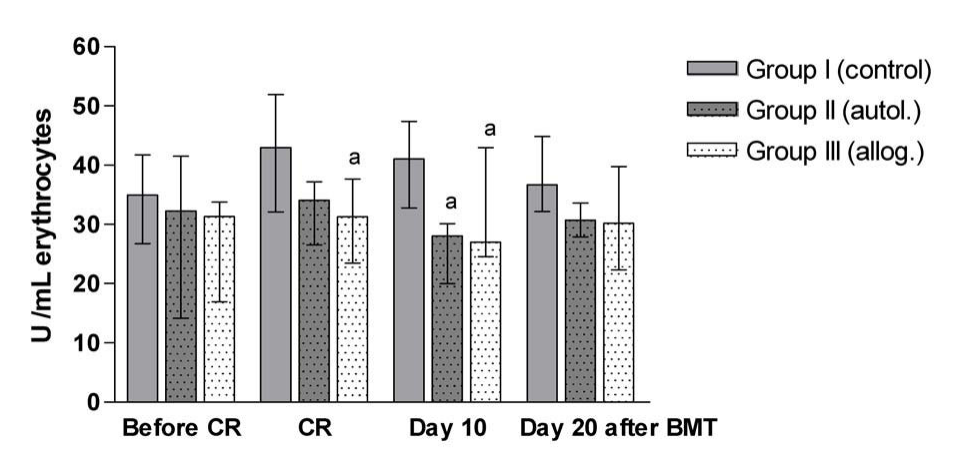
**Erythrocyte superoxide dismutase (SOD) activity over time**. CR: conditioning regimen; BMT: bone marrow transplantation. Data are expressed as median (lower/upper quartile). Group I (n = 37); Group II (n = 13); Group III (n = 24).^a ^Significantly different from group I (control).

Erythrocyte superoxide dismutase (SOD) activity was significantly lower in group II (autol.) than in group I (control), on day 10 after BMT (p < 0.0005) and was also lower in group III (allog.) than in group I (control) during CR (p < 0.05) and on day 10 after BMT (p < 0.01). Groups II and III were not significantly different from each other in erythrocyte SOD activity and enzyme activity did not change over time in these groups (Fig. 2).

### Protein thiol groups

Plasma protein thiol (P-SH) group levels were significantly lower in groups II (autol.) and III (allog.), before CR (p < 0.01), during CR and on days 10 and 20 after BMT, (p < 0.00001) when compared with group I (control). P-SH levels in groups II and III were not significantly different from each other. P-SH levels decreased from before CR to day 20 after BMT in the patients of group III and decreased from during CR to day 20 after BMT in group II (p < 0.05), (table 4).

**Table 4:** Plasma protein thiol (P-SH) group levels over time

Groups	Before CR	CR	Day 10 after BMT	Day 20 after BMT
Group I (control)	463.50 (445.20/487.80)	432.00 (406.20/469.35)	459.00 (433.76/498.00)	429.66 (403.75/451.75)
Group II (autol.)	364.51 (335.82/407.42)^a^	352.64 (306.15/371.64)^a^	304.44 (272.34/340.41)^a^	305.60 (272.21/352.03)^ad^
Group III (allog.)	407.07 (362.10/452.54)^a^	355.88 (335.25/371.10)^a^	358.37 (303.10/386.70)^a^	345.00 (308.48/383.40)^ac^

CR: conditioning regimen; BMT: bone marrow transplantation; Units – P-SH: nmol P-SH/mL plasma.

Data are expressed as median (lower/upper quartile). Group I (n = 37); Group II (n = 13); Group III (n = 24).

^a ^Significantly different from group I (control).

^c ^Significantly different from before CR.

^d ^Significantly different from CR

### Non protein thiol groups

Erythrocyte non protein thiol (NP-SH) group levels were significantly higher in group II (autol.) during CR than in group III (allog.) (p < 0.05). NP-SH levels in groups II and III were not significantly different from group I (control). Erythrocyte NP-SH levels decreased from before CR to during CR in group III (allog.) (p < 0.01), (table 5).

**Table 5:** Erythrocyte non protein thiol (NP-SH) group levels over time

Groups	Before CR	CR	Day 10 after BMT	Day 20 after BMT
Group I (control)	1933.63 (1749.00/2012.72)	1758.24 (1563.10/1909.60)	1787.09 (1535.72/1920.80)	1821.60 (1612.80/1937.60)
Group II (autol.)	2052.34 (1929.70/2084.16)	1982.40 (1716.00/2279.06)	1936.18 (1766.80/2170.30)	1741.60 (1504.00/2147.58)
Group III (allog.)	1805.06 (1664.60/2308.24)	1604.40 (1263.06/1898.24)^bc^	1748.30 (1542.29/1998.24)	1803.36 (1271.20/1932.00)

CR: conditioning regimen; BMT: bone marrow transplantation; Units – NP-SH: nmol NP-SH/mL erythrocytes. Data are expressed as median (lower/upper quartile). Group I (n = 37); Group II (n = 13); Group III (n = 24).

^b ^Significantly different between groups II (autol.) and III (allog.).

^c ^Significantly different from before CR.

### δ-Aminolevulinate dehydratase

Blood δ-ALA-D activity was significantly lower in groups II (autol.) and III (allog.) than in group I (control) during CR and on days 10 and 20 after BMT (p < 0.005). δ-ALA-D activity decreased from before CR to during CR and on days 10 and 20 after BMT in group III (allog.) and also in group II (autol.), where δ-ALA-D increased from during CR to day 20 after BMT (p < 0.05). Blood δ-ALA-D activities in groups II and III were not significantly different from each other (table 6).

**Table 6:** Blood δ-Aminolevulinate dehydratase (δ-ALA-D) activity over time

Groups	Before CR	CR	Day 10 after BMT	Day 20 after BMT
Group I (control)	4.67(3.95/5.01)	4.00(3.42/4.90)	4.10(3.80/4.60)	4.23(3.70/4.67)
Group II (autol.)	3.94(2.98/4.89)	1.78(1.20/2.52)^ac^	1.29(1.09/1.98)^ac^	2.16(1.36/2.62)^acd^
Group III (allog.)	3.67(2.14/5.52)	2.31(1.56/3.10)^ac^	1.91(1.49/2.42)^ac^	2.20(1.36/2.70)^ac^

CR: conditioning regimen; BMT: bone marrow transplantation; Units-ALA-D: nmol PBG/ml blood/h.

Data are expressed as median (lower/upper quartile). Group I (n = 37); Group II (n = 13); Group III (n = 24).

^a ^Significantly different from group I (control).

^c ^Significantly different from before CR.

^d ^Significantly different from CR

### Correlations of biochemical estimations

Statistical analysis (Spearman correlation) revealed a significant negative correlation between TBARS and P-SH and a positive correlation between SOD and δ-ALA-D before CR for all groups (Additional file 1: Panel 1, A). There was significant negative correlation between TBARS and CAT, P-SH and δ-ALA-D and a positive correlation between VIT C and P-SH and δ-ALA-D as well as between CAT and SOD and δ-ALA-D and also between δ-ALA-D and SOD and P-SH during CR for all groups (Additional file 1: Panel 1, B). There was a significant negative correlation between TBARS and VIT C, SOD, P-SH and δ-ALA-D and a positive correlation between VIT C and SOD, P-SH and δ-ALA-D as well as between CAT and SOD and between SOD and P-SH and δ-ALA-D and also between P-SH with δ-ALA-D on day 10 after BMT for all groups (Additional file 1: Panel 1, C). There was a significant negative correlation between TBARS and VIT C, P-SH and δ-ALA-D and a positive correlation between VIT C and P-SH and δ-ALA-D as well as between CAT and SOD and also between P-SH and δ-ALA-D on day 20 after BMT for all groups (Additional file 1: Panel 1, D).

## Discussion

Lipid peroxidation can cause a profound alteration in the structural integrity and functions of cell membranes and free radical-induced lipid peroxidation has been implicated in the pathogenesis of several disorders, including cancer [42]. Treatment with radiotherapy and chemotherapy has been shown to cause peroxide accumulation [9,13,43-45]. In our study, both procedures, autologous PBSCT and allogeneic BMT, were associated with higher TBARS levels during CR, and after BMT when compared with group I (control) and TBARS also showed a negative correlation with the antioxidants. However, in group II (autol.), this increase was already present before CR and there was no further increase in lipid peroxidation, probably because of the disease itself and procedures carried out previously, such as chemotherapy that precedes the collection of the patient's own bone marrow for posterior use in BMT. However, in group III (allog.), there was an increase in TBARS levels over time. This indicates that, principally in group III, both the CR and the procedure itself (BMT) were associated with an increase in lipid peroxidation.

Vitamin C is a powerful reducing agent and an important water-soluble vitamin for humans and it can scavenge O_2_^•-^, H_2_O_2_, OH^•^, aqueous peroxy radicals and singlet oxygen. Vitamin C also protects plasma lipids against lipid peroxidation and has an important role in the regeneration of α-tocopherol [46]. VIT C levels were lower in the plasma of patients from groups II (autol.) and III (allog.) than in group I (control), at all time points analyzed and VIT C also showed a negative correlation with TBARS. The CR in both procedures, autologous PBSCT and allogeneic BMT, caused a decrease in VIT C, indicating that this antioxidant defense was impaired in both groups. These results are in agreement with data published in literature [13].

Thiols fulfill important antioxidant functions in cells and biological fluids. The changes in the content of -SH groups, may indicate membrane protein damage [47]. Alkylating agents, a common group of drugs utilized in the CR, produce free radicals that can directly interact with thiol groups of proteins [17], oxidizing them to disulfides [18]. In our study, autologous and allogeneic patients presented plasma P-SH levels lower than those of healthy volunteers and also a negative correlation with TBARS, at all time points analyzed. There was also a decrease in plasma P-SH levels after the CR and BMT, demonstrating that the protein thiol groups were oxidized by cytostatic drugs utilized in the CR preceding BMT. Erythrocyte NP-SH group levels in the patient groups were not significantly different from those of the control group, however, NP-SH levels were lower in allogeneic patients (group III) than in autologous patients (group II). In group III, there was also a decrease in NP-SH levels from before CR to during CR, demonstrating that the allogeneic procedure triggered a major imbalance in this antioxidant.

As demonstrated, CAT and SOD enzymatic antioxidants were similar in the patient groups and control group before the initiation of chemotherapy or radiochemotherapy. SOD is the key enzyme required for the removal of O_2_^•- ^by converting it to hydrogen peroxide (H_2_O_2_), which can be eliminated by CAT and peroxidases. Catalase helps in neutralizing the toxic effect of H_2_O_2 _converting it to water and non-reactive oxygen species, thus it prevents the generation of hydroxyl radicals and protects cells from oxidative damage [48]. Our study demonstrated that after the transplant, SOD and CAT activity were lower in both groups II and III, when compared with the control group, at different times, and also demonstrated a positive correlation between these enzymes. This shows an imbalance in enzymatic antioxidants caused by autologous and allogeneic procedures, which together with lipid peroxidation indicates a state of oxidative stress in both situations. Thus, it is plausible to suppose that oxidative stress can exacerbate the complications in patients undergoing bone marrow transplantation.

Blood δ-ALA-D activity before the CR in both patient groups was similar to that of the control group and chemotherapy or radiochemotherapy (CR) led to a decrease in δ-ALA-D activity over time in the patient groups. δ-ALA-D is a zinc metalloenzyme, essential for all aerobic organisms, that requires reduced thiol groups for its activity [49]. Reactive oxygen species, produced during the CR [9-12] can oxidize thiol groups located inside the active site of mammalian δ-ALA-D [50,51], decreasing its activity as observed here. In fact, the decrease in δ-ALA-D activity after the CR was extremely accentuated and this occurred in all patient groups, and δ-ALA-D also showed a significant positive correlation with important antioxidants such as VIT C, CAT, SOD and P-SH and a negative correlation with TBARS, indicating that δ-ALA-D activity is a reliable marker for oxidative stress in BMT. Finally, δ-ALA-D inhibition can result in ALA accumulation, which can have pro-oxidant effects [52] and contribute to oxidative stress caused by the CR and also by the bone marrow transplantation itself. In fact, an increase of ALA is typically associated with porphyria, hereditary tyrosine and lead poisoning and its accumulation causes oxidative stress and may lead to cancer [53]. Thus, an inhibition of δ-ALA-D after the CR may contribute to cancer relapse or secondary malignancies that may arise after chemotherapy [11]. However, a decrease in δ-ALA-D could be due to the general decline of the heme route, which does not implicate ALA accumulation. To answer this question, it is necessary to study the activity of ALA-S (δ-aminolevulinic acid synthase), the rate-limiting step of heme synthesis, which could indicate whether there is, in fact, ALA accumulation.

## Conclusion

In conclusion, the results presented here indicate that some patients undergoing BMT present signs of oxidative stress even before transplantation, which are possibly a consequence of their disease or previous procedures, as is probably the case in autologous patients, which already presented increased lipid peroxidation even before the transplantation. However, in allogeneic patients this increase occurred during the CR. We also observed that the CR and the autologous or allogeneic transplantation themseles promoted modifications in the levels of non-enzymatic and enzymatic antioxidant defenses and in δ-ALA-D activity. Moreover, blood δ-ALA-D activity seems to be an additional biomarker of oxidative stress in BMT patients.

## Competing interests

The authors declare that they have no competing interests.

## Authors' contributions

TLG performed the study, carried out the assays, performed the statistical analysis and drafted the manuscript. DMB and GB carried out the assays and helped in the acquisition of data. AVF carried out the assay. JBTR helped in the statistical analysis and participated in the design and the editing of the manuscript. All authors read and approved the final manuscript.

## Pre-publication history

The pre-publication history for this paper can be accessed here:

http://www.biomedcentral.com/1471-2407/9/138/prepub

## Supplementary Material

Additional file 1 **Panel 1. correlation between biochemical estimations for all groups**. A: estimations before CR; B: estimations during CR; C: estimations on day 10 after BMT; D: estimations on day 20 after BMT. CR: conditioning regimen; BMT: bone marrow transplantation; TBARS: thiobarbituric acid-reactive substances; VIT C: vitamin C; CAT: catalase; SOD: superoxide dismutase; P-SH: protein thiol groups; NP-SH: non protein thiol groups; δ-ALA-D: δ-aminolevulinate dehydratase; n.s.: no significant. *Significantly different. Click here for file
