# Supplementary material for: δ-ALA-D activity is a reliable marker for oxidative stress in bone marrow transplant patients
Source: BMC Cancer. 2009 May 8;9:138. doi: 10.1186/1471-2407-9-138 (PMC2694815; doi:10.1186/1471-2407-9-138)
Supplement: Additional file 1 — Panel 1. correlation between biochemical estimations for all groups. A: estimations before CR; B: estimations during CR; C: estimations on day 10 after BMT; D: estimations on day 20 after BMT. CR: conditioning regimen; BMT: bone marrow transplantation; TBARS: thiobarbituric acid-reactive substances; VIT C: vitamin C; CAT: catalase; SOD: superoxide dismutase; P-SH: protein thiol groups; NP-SH: non protein thiol groups; δ-ALA-D: δ-aminolevulinate dehydratase; n.s.: no significant. *Significantly different. [file 1471-2407-9-138-S1.doc]

A

| Estimations before CR | VIT C | CAT | SOD | P-SH | NP-SH | δ-ALA-D |
| --- | --- | --- | --- | --- | --- | --- |
| TBARS | −0.027  (n.s.) | 0.074 (n.s.) | −0.015  (n.s.) | −0.447^*^ (p=0.005) | −0.057  (n.s.) | −0.052  (n.s.) |
| VIT C | _ | 0.143  (n.s.) | −0.152  (n.s.) | 0.265  (n.s.) | −0.344  (n.s.) | 0.204  (n.s.) |
| CAT | _ | _ | 0.158  (n.s.) | −0.256  (n.s.) | −0.122  (n.s.) | 0.324  (n.s.) |
| SOD | _ | _ | _ | −0.031  (n.s.) | 0.151  (n.s.) | 0.357^*^  (p=0.027) |
| P-SH | _ | _ | _ | _ | 0.069  (n.s.) | −0.102  (n.s.) |
| NP-SH | _ | _ | _ | _ | _ | −0.022  (n.s.) |

B

| Estimations CR | VIT C | CAT | SOD | P-SH | NP-SH | δ-ALA-D |
| --- | --- | --- | --- | --- | --- | --- |
| TBARS | −0.097  (n.s.) | −0.272^*^  (p=0.018) | −0.202  (n.s.) | −0.327^*^  (p=0.004) | −0.121  (n.s.) | −0.390^*^  (p=0.000) |
| VIT C | _ | −0.062  (n.s.) | −0.028  (n.s.) | 0.332^*^  (p=0.003) | −0.110  (n.s.) | 0.360^*^  (p=0.001) |
| CAT | _ | _ | 0.254^*^  (p=0.033) | 0.015  (n.s.) | 0.126  (n.s.) | 0.278^*^  (p=0.016) |
| SOD | _ | _ | _ | 0.075  (n.s.) | 0.076  (n.s.) | 0.305 ^*^  (p=0.010) |
| P-SH | _ | _ | _ | _ | −0.012  (n.s.) | 0.616^*^  (p=0.000) |
| NP-SH | _ | _ | _ | _ | _ | 0.000  (n.s.) |

C

| Estimations day 10 after BMT | VIT C | CAT | SOD | P-SH | NP-SH | δ-ALA-D |
| --- | --- | --- | --- | --- | --- | --- |
| TBARS | −0.543^*^  (p=0.000) | 0.094  (n.s.) | −0.389^*^  (p=0.001) | −0.536^*^  (p=0.000) | 0.220  (n.s.) | −0.590^*^  (p=0.000) |
| VIT C | _ | −0.067  (n.s.) | 0.375^*^  (p=0.003) | 0.601^*^  (p=0.000) | −0.021  (n.s.) | 0.610 ^*^  (p=0.000) |
| CAT | _ | _ | 0.376^*^  (p=0.001) | 0.001  (n.s.) | 0.019  (n.s.) | 0.121  (n.s.) |
| SOD | _ | _ | _ | 0.252^*^  (p=0.039) | −0.130  (n.s.) | 0.375 ^*^  (p=0.001) |
| P-SH | _ | _ | _ | _ | −0.118  (n.s.) | 0.624^*^  (p=0.000) |
| NP-SH | _ | _ | _ | _ | _ | −0.005  (n.s.) |

D

| Estimations day 20 after BMT | VIT C | CAT | SOD | P-SH | NP-SH | δ-ALA-D |
| --- | --- | --- | --- | --- | --- | --- |
| TBARS | −0.567^*^  (p=0.000) | −0.156  (n.s.) | −0.252  (n.s.) | −0.296^*^  (p=0.014) | 0.048  (n.s.) | −0.482 ^*^  (p=0.000) |
| VIT C | _ | 0.104  (n.s.) | 0.057  (n.s.) | 0.386 ^*^  (p=0.002) | −0.010  (n.s.) | 0.565 ^*^  (p=0.000) |
| CAT | _ | _ | 0.295 ^*^  (p=0.020) | −0.000  (n.s.) | −0.113  (n.s.) | 0.170  (n.s.) |
| SOD | _ | _ | _ | 0.077  (n.s.) | −0.026  (n.s.) | 0.174  (n.s.) |
| P-SH | _ | _ | _ | _ | −0.109  (n.s.) | 0.644^*^  (p=0.000) |
| NP-SH | _ | _ | _ | _ | _ | 0.034  (n.s.) |

Panel 1. Correlation between biochemical estimations for all groups. A: estimations before CR; B: estimations during CR; C: estimations on day 10 after BMT; D: estimations on day 20 after BMT. CR: conditioning regimen; BMT: bone marrow transplantation; TBARS: thiobarbituric acid-reactive substances; VIT C: vitamin C; CAT: catalase; SOD: superoxide dismutase; P-SH: protein thiol groups; NP-SH: non protein thiol groups; δ-ALA-D: δ-aminolevulinate dehydratase; n.s.: no significant.

^*^Significantly different.
